# Supplementary material for: Stakeholder Perspectives on the Acceptability, Design, and Integration of Produce Prescriptions for People with Type 2 Diabetes in Australia: A Formative Study
Source: Int J Environ Res Public Health. 2024 Oct 8;21(10):1330. doi: 10.3390/ijerph21101330 (PMC11507040; doi:10.3390/ijerph21101330)
Supplement: Supplementary file 1 [file ijerph-21-01330-s001.zip › Supplementary Material S2. Survey outline.pdf]

CONSULTATION SURVEY FOR BROADER STAKEHOLDER GROUP

Produce Prescription: innovative 'Food is Medicine' intervention to improve health among people with type 2 diabetes

Prof Jason Wu

Each stakeholder will receive the same set of open-ended survey questions

---

Background

1. **Please indicate which stakeholder category you are representing**:
   - ☐ Primary Health Network
   - ☐ Local Health District/Health Service Provider
   - ☐ Collaborative
   - ☐ Food provider
   - ☐ Education provider
   - ☐ Research partner
   - ☐ Government - Federal
   - ☐ Government – State
   - ☐ Consumer
   - ☐ Clinician/Health professional

2. **Please select the category that best reflects your role/position at your organisation:**
   - ☐ Executive
   - ☐ Management
   - ☐ Clinician
   - ☐ Policy maker
   - ☐ Academic
   - ☐ Program operations
   - ☐ Other

3. **From your personal and/or organisational perspective, do produce prescriptions align with your/your organisation's goals or values?**
   a) **If yes, why**
   b) **If no, why not?**

4. **Broadly speaking, what do you think are the:**
   a) **potential benefits of implementing produce prescription programs into the Australian healthcare system?**
   b) **potential challenges of implementing produce prescription programs into the Australian healthcare system?**

Program Design

5. **What group(s) of individuals with type 2 diabetes should a produce prescription program be designed for? Why?** *Prompts: Consider ranges and severity of health status/biomarkers and social determinants measures (e.g. food insecurity, income), access, equity*

6. **Which organisations are important to have involved in a produce prescription program for Type 2 diabetes?**
   a) **What do you think each of their roles would be?**
   b) **How should they work together?**
   Prompts: consider food vendors, healthcare, 'prescribers', nutrition education, implementing organisations, research/evaluation, insurers/funders, partnership agreements, data sharing requirements.

7. **Considering your district context/location, what would be the best way (i.e. mechanism) to get fresh produce to participants in this type of program?** *Prompts: consider program accessibility, viability, duration, infrastructure, technology, resources, governance.*

   a) **Can you think of any barriers participants may have in receiving the produce?**
   b) **Can you think of any barriers to participants using produce prescriptions?**
   Prompts: consider metro vs rural/remote differences, cultural and vendor diversity, stigma

8. **From your perspective, what kind of data, measures and outcomes would be important to capture as part of produce prescription programs, to adequately assess the impact of the program on the participants?** *Prompts: clinical markers, hospitalisations/healthcare use, fruit & vegetable intake, food security, participant retention/satisfaction, adherence to care plan/medical advice, participant self-efficacy, agency KPI measures*

9. **Who has primary responsibility for program monitoring and evaluation?**

<u>**Implementation and Adoption**</u>

Now having considered the above, we would like you to think about how you see produce prescription as a 'therapy' being integrated into a model of care for type 2 diabetes at the local / district / state level.

10. **What existing initiatives/schemes/services could this 'prescription' be integrated with to improve its sustainability and success?** *Prompts: consider coordinated care programs, state-based integrated care programs, existing prevention/treatment services.*

11. **How would screening and referral for eligible participants to this type of program be achieved?** *Prompts: consider what screening and referral options are currently available, staffing and/or technology requirements, what standardised tools could be used*
    a) **Do you think GP or other health professional referrals could work? What considerations go with this?**

12. **From your perspective, what potential funding models would support implementation of produce prescription programs in NSW and Australia?**
    Prompts: Some potential funding models to consider include:
    - Block funding
    - Collaborative commissioning – shared investment
    - National Diabetes Services Scheme (NDSS)
    - The Medical Benefits Schedule (MBS)
    - The Pharmaceutical Benefits Scheme (PBS)
    - Bundled funding.

13. **Can you think of any challenges to covering the costs?** *Prompts: Standard cost drivers include cost per service, technological set-up (e.g., POS, EMR, distribution), admin/implementation costs, vendor, other nutrition support (e.g. education) and evaluation costs.*

Conclusion

13. **Is there anything else that you feel is important to consider for sustainable produce prescription program implementation in NSW/Australia?** *Prompts: consider what legal and/or governance items are important, what policy or strategies to align with, what longer term resources and patient supports are needed.*
